# Econometric Analysis of Optimal Site of Care for Prefrail and Frail Patients Undergoing Mastectomy for Breast Cancer

**DOI:** 10.1093/geroni/igaf122.3751

**Published:** 2025-12-31

**Authors:** Claire Morton, Yu-Jen Chen, Kenneth Williams, Randall Bloch, Ezra Brooks, Christina Minami, Zara Cooper, Louis Nguyen

**Affiliations:** Center for Surgery and Public Health, Boston, Massachusetts, United States; Brigham and Women’s Hospital, Boston, Massachusetts, United States; Brigham and Women’s Hospital, Boston, Massachusetts, United States; Boston Medical Center, Boston, Massachusetts, United States; Brigham and Women’s Hospital, Boston, Massachusetts, United States; Brigham and Women’s Hospital, Boston, Massachusetts, United States; Brigham and Women’s Hospital, Boston, Massachusetts, United States; Brigham and Women’s Hospital, Boston, Massachusetts, United States

## Abstract

Patients with breast cancer undergo mastectomy in ambulatory surgery centers (ASCs) or inpatient settings, each considered safe for appropriate patients. Appropriateness remains poorly defined and relies on subjective clinician assessment contributing to variability in practice patterns that may be suboptimal. Utilizing the AHQR HCUP NIS and NASS, we sought to understand the association of frailty, defined by the Hospital Frailty Risk Score, with site of care. Secondary outcomes included transfer to an acute care hospital from an ASC and cost of care. 85.3% of all patients (190,148) and 51.3% (2,603) of prefrail or frail patients underwent surgery in ASCs. On multivariable analysis, frailty or prefrailty (OR 5.856) was associated with increased inpatient care (p < 0.001). Rates of transfer were low (0.1%). Odds of transfer were higher among prefrail and frail patients (OR 2.640, p < 0.05) but rates remained low (< 0.4%). Given clinician sorting, expected costs of mastectomy for prefrail and frail patients were greater than those for robust patients ($56,036.23, SE $396.05, vs $49,929.62, SE: $142.89). We calculate that rates of transfer that would negate cost savings associated with ambulatory procedures is over 100 times the currently observed rate (38%, SE 4.7%). If all prefrail and frail patients received care at ASCs with transfer rates at currently observed levels expected cost savings would average $8,404 per patient. Our findings suggest that despite slightly higher rates of transfer to an acute care hospital following ASC care, clinicians should consider treating frail and prefrail older adults in ASCs more often.

